# Time-warping analysis for biological signals: methodology and application

**DOI:** 10.1038/s41598-025-95108-5

**Published:** 2025-04-05

**Authors:** Aleksei Krotov, Reza Sharif Razavian, Mohsen Sadeghi, Dagmar Sternad

**Affiliations:** 1 Department of Bioengineering, Northeastern University, Boston, USA; 2 Department of Mechanical Engineering, Northern Arizona University, Flagstaff, AZ USA; 3 Department of Biology, Northeastern University, Boston, USA; 4 Department of Electrical and Computer Engineering, Northeastern University, Boston, USA; 5 Institute of Experiential Robotics, Northeastern University, Boston, USA

**Keywords:** Computational neuroscience, Motor control, Time series, Neuroscience, Systems biology, Computational neuroscience, Data processing

## Abstract

Any set of biological signals has variability, both in the temporal and spatial domains. To extract characteristic features of the ensemble, these spatiotemporal profiles are typically summarized by their mean and variance, often requiring prior padding or resampling of the data to equalize signal length. Such compression can conceal essential information in the signal. This work presents the method of time-warping, reformulated as elastic functional data analysis (EFDA), in an accessible way. This powerful approach rescales the temporal evolution of signals, aligns them accurately, decouples their spatial and temporal variability, and faithfully extracts their characteristics. This technique was compared to conventional methods of normalizing or padding data followed by averaging, using synthetized signals with controlled variability and real human data from a complex manipulation task. Comparative analysis demonstrates that EFDA successfully reveals otherwise concealed features and teases apart temporal and spatial variability. Critical advances to the more common method of dynamic time-warping (DTW) are discussed. Application of EFDA and potential new insights are illustrated in the context of human motor neuroscience. Annotated code to facilitate the use of this technique is provided.

## Introduction

Biological signals are noisy, both in amplitude and time. When repeated measurements are obtained from the ‘same’ process, the challenge for the scientist is to extract the salient features that are characteristic to the process of interest. Typically, these spatiotemporal profiles are summarized by their mean and variance, often requiring time normalization by resampling and binning of the data. However, such data compression is prone to lose, or even distort, essential information. This paper aims to present elastic functional data analysis (EFDA), a recent development of the time-warping method, in an accessible way and highlight its benefits over other conventional methods with synthetic and some experimental data.

This method has been developed in statistics and applied in computer animation and speech analysis, but only in few other domains where it may also be relevant^1–3^. One reason may be that the technique is mathematically and computationally sophisticated and several variants of it exist that can present a hurdle for a newcomer to apply the technique to their own data. This paper aims to present one recent method for time-warping alignment in tutorial fashion, together with an annotated ready-to-use code and an example application to data from motor neuroscience, where this method has found little application. Thus far, the goal of this paper is to motivate more frequent use of this data analysis technique.

### A first overview of time-warping

**Fig. 1:**
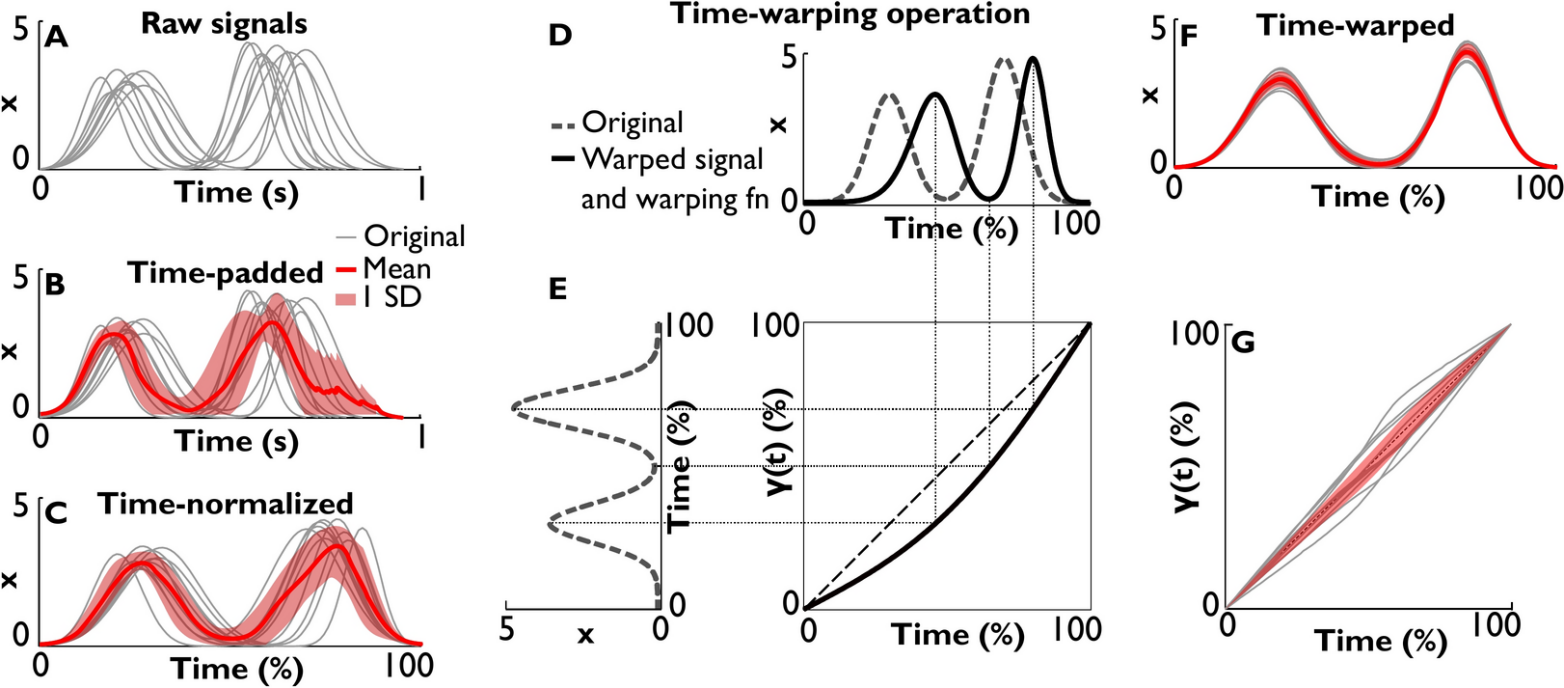
Averaging time-series with spatial and temporal variability. (**A**) Synthetic two-peaked signals generated with a Gaussian function. (**B**) Generating signals of equal length via padding NaNs at the end of the signal. Resulting mean profile and standard deviations (SD) are shown by the red line and the shaded band around the mean, respectively. (**C**) Normalizing data by resampling the time-series to equalize number of samples for all signals. Resulting mean and standard deviation (SD) are shown by the red line and the shaded band around the mean, respectively. (**D**) Example of time-warping a single signal. The original signal is shown with a dashed line, and the warped signal with a solid line. (**E**) Temporal remapping of original time to warped time *γ*(*t*). After the time normalization, both have units of percent. (**F**) Mean and SD after time-normalizing and aligning the ensemble of signals shown in panel A via time-warping. (**G**) Warping functions that emerged with the alignments. Each time of a signal before alignment (%) is mapped into its time after alignment *γ*(*t*) (%).

Research in life science typically starts with raw data that are time-series with varying amplitudes and durations. Regardless of whether the time-varying metric is displacement, force, intensity, or any other physiological quantity, the analysis problems have much in common. Figure 1A illustrates an exemplary ensemble of signals that was synthesized to have two peaks with normally distributed noise added to peak values and peak locations (see more in Section “Benefits of time-warping shown on a synthetic dataset as ground truth”). Inspection of these curves reveals common patterns and features of interest, such as maxima and minima, but with different intervals between those. Further analysis often necessitates discrete estimates of temporal and spatial features, but quantification of, for example, peak amplitude might be challenged by its varying location and shape and by the trials’ different overall durations and number of samples.

A straightforward method of averaging time-series of different durations and different number of samples is assuming a common start and padding the shorter time-series with zeros or non-numerical numbers (NaNs) to obtain the same number of samples in all signals, as shown in Fig. 1B. The mean and standard deviations may be then readily calculated at every time point across the entire signal, rendering the mean time-series in red with the shaded band indicating standard deviations at every time point. This averaging procedure not only masks or inflates the salient features, but may even create false features that were not present in the original signals (the red averaged signal created an additional small peak at the end).

Evidently, when padding the time-series, the number of samples in the later portions of the time-series decreases and confounds the calculation of the means in these portions. Therefore, a second, more frequently used method is assuming both a common start and end, normalizing the different trial durations to 100% and re-sampling the data uniformly across the different realizations (Fig. 1C). The means and standard deviations calculated at every time point then exhibit a different summary of the spatiotemporal evolution. The example here shows a slightly distorted and slower rise to the second peak, compared to the individual time-series and the zero-padded mean. This is the typical case resulting in underestimating peak values and overestimating peak widths.

In addition, variability estimates may also become unreliable. Spatial variability is readily assessed by examining standard deviations of salient features, such as amplitude peaks; temporal variability is assessed by standard deviations of movement time or intervals between chosen landmarks. The variability of an ensemble of time-series is also estimated by the standard deviations at every time point integrated over the time-series, as shown by the shaded band around the mean in Figs. 1B and C. This can be summarized by the sum-of-squares or root-mean-square error (RMSE). However, this latter estimate is critically influenced by the signal’s temporal profile and duration, because the relevant peaks and valleys may be misaligned.

Alignment of the features of interest, or registration of curves, was developed in statistics and is based on the concept of re-parametrization. Time-series are a special case of one-dimensional curves, and their re-parametrization is a mathematical expression of their temporal (horizontal) deformation. Figure 1D illustrates how a two-peaked time-series (black solid line) can be re-parametrized or ‘time-warped’, i.e., stretched and compressed in the time domain (grey line), without affecting the amplitude. Figure 1E illustrates the warped time: as regular clock time progresses linearly (dashed line), the warped time $\gamma (t)$ (solid line) first progresses slower, then faster than the original clock time. Figure 1F illustrates how alignment by time-warping may better summarize the time-series in Fig. 1A and extract salient features and their variability. The time-series are now dilated or compressed according to the signal’s shape. Figure 1G shows the temporal transformations of all signals as a collection of warping functions $\gamma (t)$ (grey lines) with their mean in red and standard deviation in the pink shade.

Several methods for warping time-series have already been developed^4,5^. The classical approach that is still widely used is ‘dynamic time warping’ (DTW) that relies on minimizing Euclidean distances between the time-series^1,6^. More recently, elastic functional data analysis (EFDA) was developed that uses a more advanced mathematical framework^5,7,8^. EFDA has several advantages over dynamic time warping, including the ability to decouple spatial and temporal variability and maintain continuity of the signals (one critical weakness is “pinching” explained in Appendix II). EFDA was demonstrated in several studies on neural protein shapes, neural spike trains, and shoe sensor data^8–10^. While DTW is available as a built-in function in the commonly used software packages, EFDA is much less known, but has critical advantages. This paper reviews curve registration of time-series by EFDA, followed by an application to kinematic data from human movements. Given some overlap of terminology in the literature, we invite the readers to refer to the definitions adopted in this work (Appendix I).

The first section introduces the technique of EFDA for the optimal alignment of time-series and illustrates it on a synthetic dataset. The second section compares EFDA alignment with two conventional approaches on a synthetic dataset, where ground truth is known. The third section demonstrates how EFDA is applied to experimental data of humans manipulating a whip and discusses the insights possible when using the method.

## Alignment via time-warping and estimates of spatial and temporal variability

To optimally align a set of time-series from the same process, continuous time-warping alignment may be applied, also known as elastic curve registration^4,5^. For time-series defined on the real domain of linearly scaled, normalized time $t\in \left[\text{0,1}\right]$, the (time-)warping function *γ*(*t*) is a continuous and monotonically increasing mapping of the domain [0, 1] onto itself. The time-warping operation, written as a re-parametrization *f*(*t*)◦*γ*(*t*) = *f*(*γ*(*t*)), results in a non-uniform temporal scaling of the original signal (Fig. 1E,G). Such scaling could be viewed as a continuous adjustment of the “speed” of time, or the “internal clock”, in contrast to the linearly progressing external clock time.

### Aligning signals: calculating the distance between two or more signals

To find the optimal warping function for alignment, it is necessary to first quantify and then minimize the distance between two time-series *x*_*1*_(*t*) and *x*_*2*_(*t*). The EFDA approach uses the Fisher-Rao distance *d*_*FR*_^11–13^. Calculating *d*_*FR*_ involves two steps: First, the time-series *x*_*i*_(*t*) are transformed into a square root rate function (SRRF):

$$q_{i} (t) = \frac{{\dot{x}_{i} (t)}}{{\sqrt {|\dot{x}_{i} (t)|} }}$$

This transformation considers the derivatives, or slopes of the orignal signal to balance stretching or compressing time with maintaining continuity of the signals (see more details in Appendix II). The second operation calculates the Fisher-Rao distance *d*_*FR*_ between two transformed signals *q*_*1*_(*t*) and *q*_*2*_(*t*) by computing the L2 norm:

$$d_{FR} (x_{1} ,x_{2} ) = d_{L2} (q_{1} ,q_{2} ) = \sqrt {\int_{0}^{1} {\left( {q_{2} (t) - q_{1} (t)} \right)^{2} dt} }$$

Some advantages of this distance metric are that it results in the same value regardless of alignment direction (see below) and that it prevents over-stretching and over-compressing portions of the signal (“pinching”, see Appendix II on the comparison of the approaches)^12,13^.

**Fig. 2:**
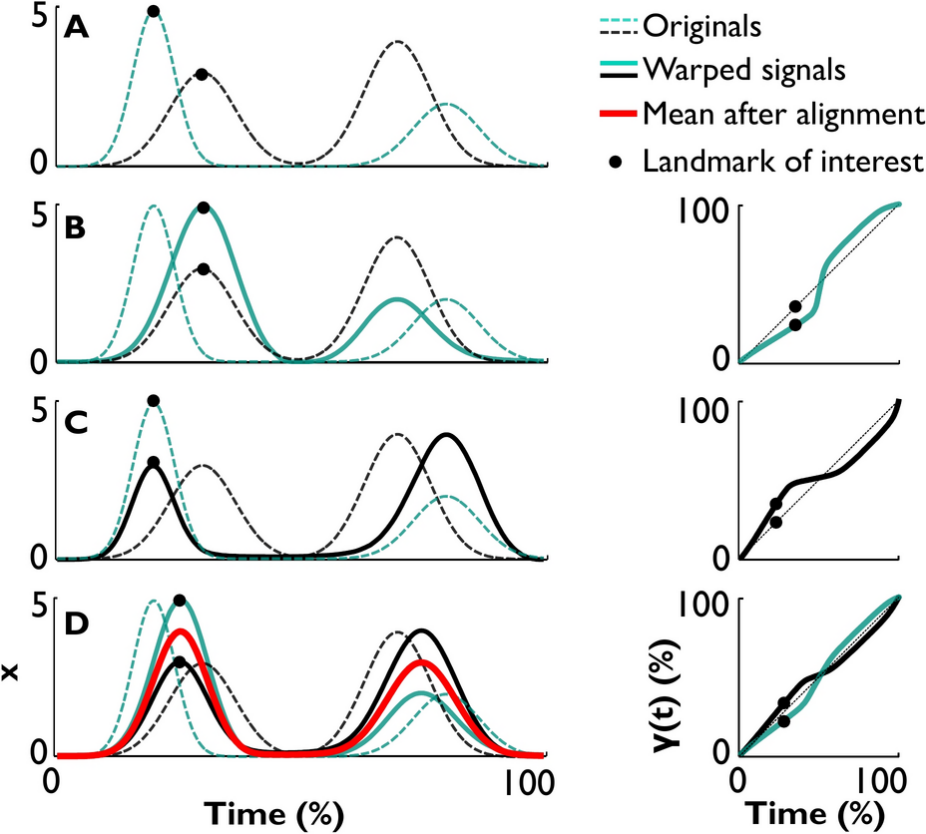
Alignment of signals via time-warping. (**A**) Two synthetic signals with similar two-peaked structure. An exemplary landmark of interest is the first peak marked with black dots in both signals to highlight the misalignment in time. (**B**) Alignment via time-warping of the green dashed signal to the black dashed signal rendering the green solid signal. The corresponding warping function is on the right, the black dots showing the temporal alignment of the peaks. (**C**) Alignment via time-warping of the black dashed signal to the green dashed signal. The corresponding warping function is shown on the right. (**D**) Simultaneous alignment of both signals to render a mean signal shown in red. The corresponding warping functions are shown on the right, again highlighting the temporal alignment of the first peaks.

Alignment of time-series is illustrated in Fig. 2A on two exemplary signals with similar spatiotemporal structure, but with visibly misaligned peaks. Their alignment is achieved by minimizing *d*_*FR*_ between them while using one or the other as template, or warping both signals to arrive at an emerging template. Figure 2B shows the alignment of the green dashed time-series using the black dashed time-series as the template, generating the green solid curve. Figure 2C displays the signals using the green dashed time-series as template for the black dashed curve, leading to the black solid curve. The two time-series may also be simultaneously aligned to an emerging common template that then represents their mean shape, as shown by the red line in Fig. 2D. The optimal template is defined and numerically approximated with the two goals^11^: (1) to have the smallest sum of *d*_*FR*_ between the template and the set of original time-series, and (2) to result in time-warping functions whose mean is identity $\gamma (t)$ = *t* (see Fig. 1G). During each of the three directions of alignment, *d*_*FR*_ between the signals achieves the same minimum value (see further discussion of distance metrics in Appendix II). To minimize *d*_*FR*_ and align the signals, several optimization approaches have been used, including dynamic programming and stochastic gradient descent^7,14^. These optimization techniques are not specific to time-warping. Our code used dynamic programming as it was simpler for the example datasets. The code for the analyses is provided as Matlab functions for download on a public repository EFDA_MovementSignalAlignment^15^.

Note that all three cases in panels B, C, D generate different aligned pairs of signals with different means and different warping functions. Which one of the three alignment directions is chosen is dictated by the scientific question. For each of the three alignment directions, the time-warping functions are plotted on the right of each panel. The black dots highlight that the two peaks are coincident after warping one or both time-series. They also indicate the amount of temporal shift of those peaks on the warped time axis. Importantly, the minimized *d*_*FR*_ reflects only the amplitude or shape difference between the aligned signals; it is decoupled from the signals’ temporal “elastic” differences^5,8^. The latter reflect how much the signals need to be warped for alignment and are defined in the warping function domain, separate from the spatial domain.

### Separating spatial and temporal aspects of variability

**Fig. 3:**
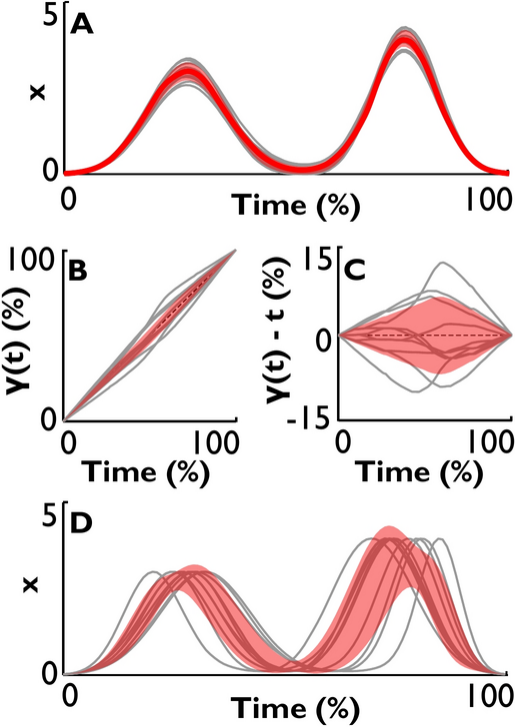
Extraction of spatial and temporal variability following time-warping alignment. (**A**) Mean time-series and standard deviations (red line and shaded band) after time-normalizing and aligning the signals via time-warping. (**B**) Warping functions resulting from the alignment; they map normalized time of each signal segment after alignment to its normalized times before alignment. (**C**) Enlarged display of the warping functions with unity subtracted. (**D**) Warping of the aligned mean time-series (solid line in **A**) with every warping function (see **B**); the standard deviation band visualizes the contribution of temporal variability to the spatial domain.

After alignment of multiple time-series, the signals continue to exhibit variability in the amplitude domain (Fig. 3A). To quantify this spatial variability in the aligned time-series*,* the standard deviations across signals at each time point are summed across time, which is an intuitive, numerically accurate proxy for RMSE:

$$Var_{Spat} (X^{N \times M} ) = \frac{1}{N}\sum\limits_{{}}^{N} {SD_{M} (x)} ,$$

for an ensemble *X* of *M* signals resampled to *N* time samples. This measure keeps the same units as the original data, e.g., centimeters for a position signal. In addition, the warping functions found during alignment allow to separately quantify temporal variability of the ensemble. Temporal variability is standard deviations across the warping functions at each time point summed across time,

$$Var_{Temp} (\Gamma^{N \times M} ) = \frac{1}{N}\sum\limits_{{}}^{N} {SD_{M} (\gamma )} ,$$

for an ensemble Г of the warping functions obtained during alignment of *X*. The standard deviations around the mean warping function (unity *t *= *t*, up to numerical precision)  can be calculated in the original mapping space or after subtracting the mean warping function from all individual functions (Fig. 3B,C). Temporal variability has arbitrary units, due to normalized time.

A third type of variability quantifies the contribution of temporal variability to the spatial domain. To calculate this variability, the mean of the aligned time-series $< x >$ is warped with each of the ensemble’s warping function $\gamma_{j}$ separately. This creates a new ensemble *X*^*w*^ of *M* time-series that all have identical “shapes” and amplitudes, but varying temporal evolution (Fig. 3D). Integrating across time the standard deviations across the time-series:

$$Var_{Temp2Spat} (X,\Gamma ) = \frac{1}{N}\sum\limits_{{}}^{N} {SD_{M} (x^{w} )} ,$$

becomes an estimate of the spatial variability due to temporal variability in the original ensemble, expressed again in the units of original signal.

To further illustrate the benefits of alignment with time-warping over time-padding and time-normalization, the calculations of the different kinds of variability are demonstrated on an extended synthesized dataset. Here, the function that generated the synthetic dataset is used to also calculate the true variability, i.e., ‘ground truth’, that can be compared against the estimated variability.

## Benefits of time-warping shown on a synthetic dataset as ground truth

We synthesized an ensemble of signals with a known Gaussian function with three landmarks, two peaks and one valley, and different sources of noise. We applied time-padding, time-normalization, and time-warping and estimated the discrete parameters of the time-series, i.e., maxima and minima and their means and standard deviations. These results were compared with the known parameter values obtained from the synthesized noisy signals.

**Fig. 4:**
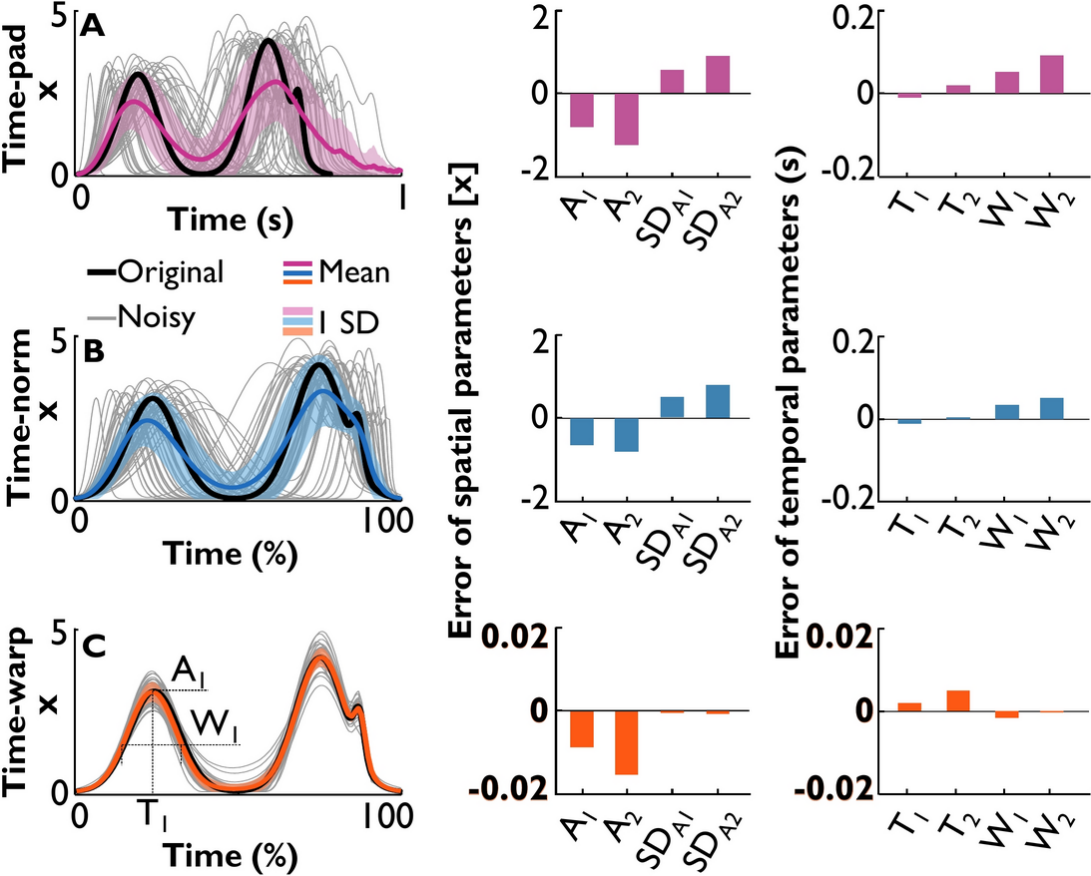
Comparison of the three approaches on a set of synthetic signals with noisy parameters (two peak heights, widths, and locations). (**A**) Time-padding approach, equalizing the signal lengths by padding NaNs at the end of the shorter signals. (**B**) Time-normalization approach, equalizing signal lengths by uniformly rescaling time. (**C**) Time-warping approach: after time-normalization the signals are warped non-uniformly. The first column shows the noise-free original signal in black; the grey lines are the signals generated by adding noise to the function parameters; the colored line with its shaded band is the mean signal and its standard deviations, generated by each method. In the second and third columns, errors between the feature estimates from the mean time-series and the distributions of the estimates from each aligned noisy signal. The second column displays amplitude-related features (peak amplitudes, their standard deviations), the third column displays time-related features (amplitude peak locations and peak widths). Note the 100-fold and 10-fold scale differences in the second and third columns of (**C**).

Figure 4A shows 500 signals generated by the triple-Gaussian function:

$$\begin{aligned} x(t) & = A_{1} \exp \left( { - \frac{4\ln 2}{{W_{1}^{2} }}(t - T_{1} )^{2} } \right) + A_{2} \exp \left( { - \frac{4\ln 2}{{W_{2}^{2} }}(t - T_{2} )^{2} } \right) \\ & \quad + \frac{{A_{2} }}{3}\exp \left( { - \frac{4\ln 2}{{(W_{2} /5)^{2} }}(t - T_{2} - W_{2} /2)^{2} } \right), \\ \end{aligned}$$

on the domain $t\in \left[\text{0,1}\right]$, where *A*_1_ and *A*_2_ are the peak amplitudes, *W*_1_ and *W*_2_ are the full widths at half-maximum, and *T*_1_ and *T*_2_ are the times of the peaks. Note that the third term in the equation used the scaled parameters of the second peak, *A*_2_, *T*_2_, and *W*_2_, and defined an additional small and narrow peak. Although its parameters were not additionally estimated, this created a relatively small, but salient landmark for qualitative examination. The parameters used for the synthetic data are summarized in Table 1. To impose a controlled amount of variability, zero-mean Gaussian noise was added to all six parameters. For the subsequent quantifications, the onsets and offsets were defined at 2% of the maximum peak value. A similar set of functions, but without the third small peak, was used for illustrations in the previous figures.

**Table 1:** Parameter values used for generating the synthetic data.

	Original	Added noise (σ)
A_1_	3	0.3
A_2_	4	0.3
B_1_ (s)	0.16	0.05
B_2_ (s)	0.16	0.05
ΔT (s)	0.4	0.03
t_0_ (s)	0.3	–
Onset/offset (%max)	2	–

The original values for the Gaussian function, the number for the added noise are one standard deviation of a Gaussian distribution.

To apply the three methods, the number of data samples had to be identical for all time-series. To this end, the first method aligned the onsets of all signals and padded missing values with NaN (Fig. 4A). The second method resampled the signals uniformly from 0 to 100% to generate the normalized time (Fig. 4B). The third method also started with the same resampling to 100%, but continued with the time-warping alignment (Fig. 4C). The alignment was performed with the function *fdawarp.timewarping* of the MATLAB package *fdasrvf*^7,14^ with the following settings: no box-filter smoothing, dynamic programming method for optimization, 3 maximum iterations, the smoothness parameter set to 0.01^15^. After this step, the average and standard deviation time-series were determined. The original signal, the noisy signals, and the obtained means and standard deviations of the three methods are shown in the first column of Fig. 4. As to be expected, the peaks in the mean time-series obtained via time-padding (purple line) and time-normalizing (blue line) approaches became less pronounced and wider than those obtained by time-warping (orange line) or in the original signal (black line).

For each of the 500 synthesized time-series, the amplitudes *A*_1_ and *A*_2_, peak times *T*_1_ and *T*_2_, and full-widths at half-maxima of the two peaks *W*_1_ and *W*_2_ were used to calculate the true means of all six parameters and, additionally, the standard deviations of the peaks, *SD*_*A*1_ and *SD*_*A*2_. The same eight parameters were estimated in the mean time-series resulting from each of the three approaches. The estimation error for each parameter was computed as the difference between the values obtained from the three approaches and from all simulated time-series.

The errors for spatial and temporal parameters are summarized in the bar charts on the right of the time-series in Fig. 4. Relatively large errors in time-padding and time-normalizing arose from underestimating the peak values, *A*_1_ and* A*_2_, and consequently overestimating the standard deviations of peak values, *SD*_*A*1_ and *SD*_*A*2_. In contrast, the time-warping approach demonstrated a more robust extraction of the mean time-series, with almost 100 times more accurate parameter estimates (note the reduced scale of the y-axis for time-warping errors). The right column of Fig. 4 summarizes the temporal parameters: while *T*_1_ and *T*_2_ were almost similarly accurate across all three approaches (purple, blue, and orange bars), the peak widths *W*_1_ and *W*_2_ were estimated about 10 times more accurately by the time-warping approach. Note that the tiny flick on the right of the second peak washed out with the time-padding and time-normalizing, but resolved with time-warping. The time-normalizing approach also showed an additional widening of the second peak.

**Fig. 5:**
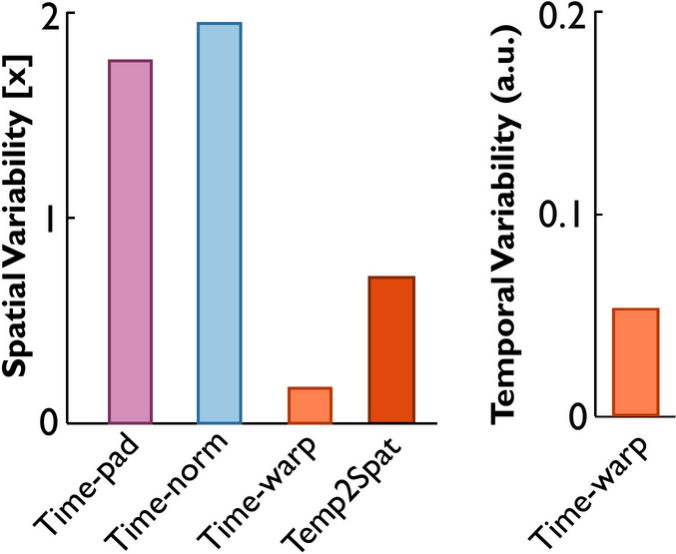
Variabilities of the same ensemble of synthetic noisy signals after applying the three alignment approaches with subsequent calculation of the mean profile and standard deviations across time points. Each variability is half of the area of the standard deviation band. Spatial variabilities were computed in the spatial domain and are in the signal’s units (arbitrary for the synthetic dataset). Temporal variability was computed in the domain of the warping functions and has units of normalized time.

In addition to these discrete parameter estimates, Fig. 5 summarizes the variability of the entire signal ensemble. Spatial variability was computed for the three methods by integrating the standard deviation across the aligned signals. Only time-warping allowed additional estimates of temporal variability and the contribution of temporal to spatial variability. Spatial variability extracted by time-warping was much smaller than by time-padding and time-normalizing, reflecting only the variability from the noise in the heights of the peaks and the valley. The time-warping approach also separated the temporal variability resulting from varying peak widths and times. Its temporal-to-spatial contribution indicated that the temporal misalignment of peaks and varying rates contributed more to the originally observed variability than did the variability due to varying peak heights. The temporal variability will be further discussed in conjunction with our experimental dataset.

## Applying time-warping to complex real movements

To demonstrate the benefits of time-warping alignment by EFDA, it was applied to real experimental data from a complex motor task collected to address the question how humans control a flexible object, such as a whip^16–19^. With the advances of recording methods, there is increasing interest in motor neuroscience in more complex motor tasks, where multiple variables and their relations are needed to provide the desired insights^20^. Variability in such data has become recognized as an important manifestation of underlying control challenges and priorities^21,22^. However, analysis of these data is difficult and estimating means and variability becomes difficult^23,24^. Yet, time-warping has not yet been applied for addressing motor control problems, with very few exceptions^25,26^. We want to demonstrate how time-warping alignment can enhance understanding of highly variable data, here exemplified by the unconstrained manipulation of a 1.6-m long bullwhip, i.e., the control of an underactuated object.

For this demonstration, we used previously reported data of two novices^17^ and a professional whip performer^27^ hitting a target with a whip in our laboratory. The experimental procedure was approved by the Institutional Review Board of Northeastern University in accordance with the ethical requirements the university and with the Helsinki Declaration and its later amendments. All participants signed an informed consent form.

**Fig. 6:**
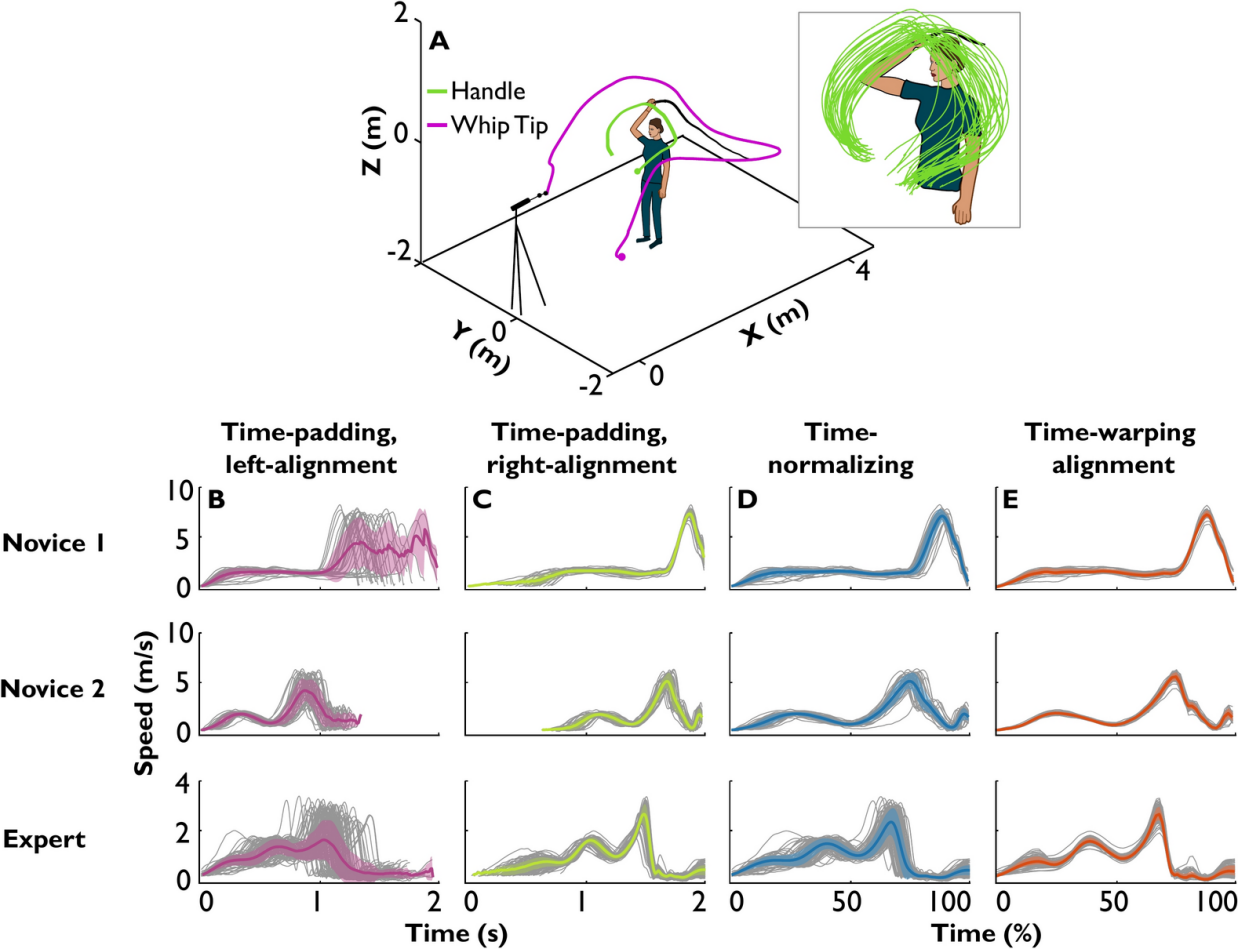
Hand kinematic data from human participants hitting a target with a bullwhip. (**A**) Experimental layout with exemplary trajectories of the right hand (green) and the tip of the whip (magenta). Inset: trajectories of the hand from 30 consecutive trials. (**B**) Hand speed aligned by the start of the trial (grey) and extracted mean and SD (purple) from two novices and the expert. Number of samples was equalized across the ensemble via padding the ends with NaNs. (**C**) Hand speed, aligned by the end of the trial (grey), and extracted mean and SD (green). The starts were padded with NaNs. (**D**) Hand speed with each time-series resampled (time-normalized) to the same number of samples and extracted mean and SD (blue). (**E**) Hand speed with the time-series normalized and aligned via time-warping and extracted mean and SD (orange).

Movements from the participants were recorded with 3D motion capture at 500 Hz (Qualisys, Gothenborg, Sweden), using one reflective marker attached to the right hand and another marker on the tip of the whip. Figure 6A shows a 3D view of an exemplary novice moving the whip (shown in purple) and aiming to hit a target. The green lines in the inset show the hand trajectories that evidently demonstrate significant variations. Each attempt to hit the target was followed by a pause. Each participant performed approximately 160 whip throws. The raw data were filtered via 4th-order Butterworth filter at cutoff frequency of 20 Hz. Figure 6B–E shows 30 time-series (grey lines) of the tangential hand speed of the two novice participants and the expert who scored 6%, 19%, and 90% target hits, respectively.

In Fig. 6B, the data were aligned at the beginning of each trial defined when the hand speed first exceeded 0.5 m/s while the distal part of the whip still laid on the floor. The set of hand trajectories exhibited a similar peak, but also substantial variability. Averaging the speeds after time-padding expectedly flattened this peak, while indicating large variability. Figure 6C shows the same original data, but now aligned at the moment when the whip was the closest to the target, marked as zero to the right of the time-series. This second type of time-padding resulted in a mean that better reproduced the shapes of individual signals and showed lower variability. Time-normalizing and averaging the data in Fig. 6D resulted in a mean and variability that were comparable to those obtained with the right-aligned time-padding method. In contrast, time-warping alignment in Fig. 6E resulted in even sharper means with noticeably decreased variability. An additional small peak after the main peak in novice 2 and in the expert, that was also present in the original data, remained visible but disappeared in the other methods.

The three conventional methods do not display consistent variability differences between the novices and the expert. For example, left-aligned time-padded Novice 1 appears just as variable as the expert (Fig. 6B) and the time-normalized time-series suggests the largest peak variability in the expert (Fig. 6D). Right-aligned time-padding suggests the best alignment among the three conventional methods, but renders unreliable trajectories at the starts of the signals due to uneven sample sizes (Fig. 6C).

Time-warping highlights elaborate features of the trajectories, particularly in the expert, where three peaks indicated bringing the hand behind, lifting the hand, then throwing the whip towards the target. The first two moves were blended together in the novices’ task execution. Although the hand speeds differed between the participants, e.g., peak speeds estimated from individual trials were 7.8 ± 0.4, 5.5 ± 0.4, and 2.8 ± 0.3 m/s, none of the alignment methods showed a corresponding effect on amplitude variability.

**Fig. 7:**
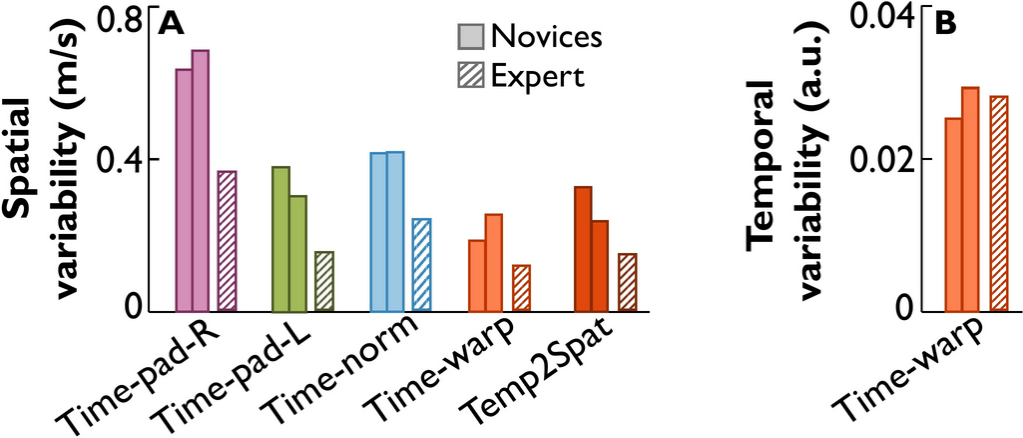
Comparison of variability estimates of hand speed between two novices (bars with solid fill) and the expert (bars with hatched fill). (**A**) The five groups show spatial variabilities for the two novices and the expert, estimated from the ensembles in the previous figure. The right-most group shows the temporal contribution to spatial variability for time-warping. (**B**) Temporal variability for the two novices and the expert estimated from the warping functions.

The hand speed from the three participants were also analyzed with all methods to extract spatial variabilities, as well as temporal variability and its spatial contribution for the time-warping approach. Figure 7A shows spatial variability estimates for the two novices (solid-colored bars), and the expert (hatched bars). All methods suggest little differences between the two novices and an expectedly lower variability in the expert. This contrasts with temporal variability shown in Fig. 7B. As the expert does not differ from the novices in temporal variability, his temporal to spatial contribution is still lower than in the novices.

### Lessons from the whip data

While the expert’s temporal contribution to spatial variability was considerably lower than that in the novices, their temporal variabilities were similar. This suggests that the expert still varied the execution rate of the hand movement, but these variations were either not relevant to the task, or, in fact, benefited the expert’s performance. Both the mean time-series and its standard deviation after time-warping alignment also revealed a subtle “flick” after the main peak at about 90% of the trial time. This was likely at the end of the whip unfolding resulting from the reflected wave as our previous studies indicated^17,28^. The flick occurred within 100 ms before the whip reached the target and is unlikely to be useful for feedback-based correction in the current trial. However, it may benefit preparing for the next trial by providing haptic information about the whip’s spatial position and direction when fully unfolded. 

## Discussion and perspectives

Conventional estimates of variability tend to be confined to quantifying spatial variability, leaving temporal ‘noise’ or variations intact. However, if variability is due to temporal shifts, a cross-sectional average taken at each time point may blur subtle features of the signals. To address this issue of misaligned time-series, time-warping assumes that each trial has its own varying execution rate or a nonlinear time evolution compared to external or measured time. Decomposing the observed variability into temporal and spatial components may offer new scientific insights.

This paper aimed to provide a tutorial-like exposition of time-warping alignment and how it can faithfully reveal informative features in an ensemble of time-series with spatiotemporal variability. While the concept itself is not new and has been used in speech analysis^1^, pattern recognition^29^**,** and operations research^30,31^**,** it is still relatively under-utilized in many other areas of science. Focusing on the EFDA method by Srivastava and colleagues^11^, we show how temporal alignment of signals can also tease apart variability in the spatial and temporal domains. This may for example be useful in motor neuroscience, where variability has already been recognized as an important source of insight^21,22,32^. This paper aims to present this sophisticated technique in an accessible fashion and provides an annotated code to facilitate its application^15^.

To demonstrate the method and its benefits over two common approaches, time-warping was applied to a synthetic dataset where all parameters, including noise levels, could serve as ground truth. Time-warping showed a clear advantage over time-padding and time-normalization: The landmark estimates obtained from the mean and the standard deviation after time-warping resulted in significantly smaller errors. To demonstrate the usefulness of this approach to motor neuroscience, we applied the method to a dataset from humans manipulating a whip and showed how fine-grained features in the time-series were revealed.

### Interpretation of decoupled variabilities

A novel use of EFDA is to decouple spatial and temporal variabilities and quantify the contribution of the temporal to the spatial domain. For example in the synthetic dataset, one (hypothetical) insight could be that the variability of the ensemble was much more affected by the temporal shifts and varying rates, than by its varying peak heights. In contrast, the experimental hand speed was affected to a similar extent by the spatial variability from varying peak heights and by the temporal variability from varying execution rates, as observed in each of the three participants. The temporal variability was similar between the two novices and the expert suggesting its pertinence to the human motor system without affecting the task outcome. In fact, the expert’s high temporal variability may indicate its reduced relevance for the task. Instead, the expert may continuously adjust the hand speed in relation to the perceived configuration of the whip, and thereby tune into the object dynamics^17,18,28^. While more studies are needed to support this interpretation, it demonstrates the new kind of questions that arise by decoupling spatial and temporal variabilities.

### Differences between time-warping approaches

The concept of time-warping where Euclidean distances between signals are minimized via dynamic programming (DTW) dates back a few decades to seminal works in speech recognition and computer animation^1,34,35^. However, several modifications have emerged since then, including the approach of Elastic Functional Data Analysis (EFDA) discussed in this work. The common concept shared between various approaches is time-warping, or re-parametrization of a curve, which is a nonlinear temporal stretching or shrinking of time^4,36^. Such re-parametrization has the purpose of aligning two or more signals, called curve registration when generalized to higher dimensions (see terminology in Appendix I). The differences between various alignment approaches arise from the choice of (1) landmark-based or continuous alignment, (2) optimization algorithm, and (3) the distance metric between two signals.

1. *Alignment* can be based on preselected landmarks, such as extrema or inflection points, or can use the entire signal in an automated fashion. In the first case, a warping function is constructed by interpolating across the landmark locations via lines (piece-wise time-normalization), polynomials, or B-splines, that are then used for alignment^37–39^. This control over temporal locations of the landmarks is computationally efficient, but may produce discontinuities and artefacts and, hence, may require additional constraints^40–42^. To avoid these disadvantages, alignment can also be continuous, i.e., include every sample of the signals as demonstrated in the elastic functional data analysis presented here. This procedure does not provide explicit control over the landmarks and requires more advanced optimization procedures, such as minimizing the Fisher-Rao distance through iterative time-warping as in the method adopted in our work. Specific applications may benefit from combining semi-manual selection of landmarks and the following time-warping of the segments between the landmarks^39,43^.
2. *Optimization* supports continuous registration, i.e., finding a warping function that would minimize a distance metric between the specified template and the warped signal. Optimization is most frequently performed via dynamic programming^44^ which guarantees an optimal solution; it is also adopted in this study and in the provided code^15^. Alternatively, optimization can also be performed using gradient descent techniques that may reduce computational cost for larger datasets, although the technique requires more tuning for appropriate application^7,45^.
3. *The distance metric* between two signals is the most important issue in the continuous registration approaches. A choice of this distance metric affects how the landmarks are aligned and what type and degree of elastic distortion is introduced to the signals. While the L2-norm (Euclidean distance) between the signals has been used most often, the EFDA framework relies on the Fisher-Rao distance. This *d*_*FR*_ distance is more sophisticated mathematically than the Euclidean distance, which improves the alignment procedure. For example, conventional dynamic time warping, relying on the Euclidean distance metric, requires additional constraints to reduce alignment artefacts. Choosing and tuning those constraints may be cumbersome. As *d*_*FR*_ prevents most of such artefacts, EFDA alignment needs only one optional smoothing parameter^10,46^. Our variability estimates from the two datasets showed little sensitivity to that parameter as long as it was small enough so that the signals could be aligned visually well. We provide a more detailed review of distance metrics in Appendix II.

### Considerations, caveats and perspectives

Warping of the time–space continuum can create interesting new problems and questions. Consider two similar displacement signals with different durations and apply conventional time-normalization dilating one signal and shrinking the other one. This raises the question whether the velocity of the dilated signal should be reduced proportionally to keep the total distance travelled invariant, or whether the velocity values should be kept invariant, essentially changing the total distance travelled. A similar issue arises with time-warping alignment. When the time-series represent velocity, as in our experimental example, alignment keeps its values invariant but changes the acceleration values (derivatives) and the cumulative distance travelled (integral). Depending on the research question, alignment may instead be applied to the time-series of the cumulative distance travelled or to the actuation forces, i.e., to its integrals or derivatives^5^.

Particular caution is also needed when studying variables with more than one dimension but of the same units, for example movements in two or three dimensions. While our study adopted tangential velocity (speed) for analysis, which is a one-dimensional signal created from a 3D movement trajectory, different coordinates (x, y, z) may also be selected for alignment. Additionally, the three coordinates may be aligned simultaneously, i.e., producing the best fitting single warping function^7,11,14,30^. These decisions affect the different variability components and their interpretations^25^.

A similar challenge presents itself in datasets with signals of multiple modalities, such as coordinates of kinematic signals, ground reaction forces, and muscle activations. One could certainly treat different modalities just like different dimensions. However, this requires data-driven or hypothesis-driven decisions about how to interpret and emphasize contributions from different modalities. To maintain ease of use even for first-time users of time-warping, we have configured our software implementation for time-series of a single variable type.

Another challenge may arise when frequencies in the variables of interest and those of the noise are close to each other. As the algorithm is sensitive to the slopes, noise might be inadvertently aligned and thus amplified. For the case of high-frequency noise and lower-frequency features of interest, low-pass filtering followed by down-sampling and aligning the data would allow obtaining smooth warping functions unaffected by noise and additionally decrease the computational burden of the alignment.

One final comment is on the possibility of more fine-grained analyses of the obtained values of the distance metric *d*_*FR*_ minimized after alignment, instead of considering variability of the ensemble. The value of this minimized *d*_*FR*_ represents the spatial difference or similarity of those signals after the temporal difference was removed. In an analogous fashion, *d*_*FR*_ can be calculated between the obtained warping functions, also in terms of their SRRF (for more detail see^5,7,47^). Such *d*_*FR*_ represents the temporal difference phase (in our one-dimensional case, the temporal) difference and may capture how much warping is needed to align one signal to another. With a view to our experimental data, where participants performed the same movement repeatedly, both those metrics decreased over consecutive trials, reflecting convergence of the hand speed to individual stereotypical patterns. Movement research may benefit from using *d*_*FR*_ to compare and quantify signatures of specific individuals and populations, such as post-stroke patients^9,26^.

### Applications in human movement research

Time-warping alignment and specifically the EFDA framework based on the Fisher-Rao distance have only been used in few isolated studies in human movement research. For example, a study that examined whole-body movements in a manufacturing environment used EFDA to compare execution rates across actions and actors and isolate principal variations in the data to suggest best practices^30^. In human gait biomechanics, EFDA was employed to align ensembles of either joint angles, ground reaction forces, or muscle forces to study within-participant and inter-participant variability^9,48^. Wang and colleagues and Swaminathan and colleagues employed time-warping alignment on joint angle and ground reaction force data for quantifying the severity of gait impairment in clinical populations^26,49^. One study on planar reaching while avoiding a bar-like obstacle summarized multiple repetitions by quantifying variability within and between participants and conditions^25^.

One likely reason why time warping has not been used more in motor neuroscience may be because the focus has been on simplified movements, where time was strictly controlled or temporal variability was not of particular importance. There is a growing body of motor neuroscience research that has turned to more naturalistic skills, where kinematic data become significantly richer due to the number of involved body degrees of freedom and the interaction with objects or the environment^17,23,24,50,51^. The study of such complex tasks has become feasible with significant progress in data recording techniques, analysis methods and, of course, higher computing power. Time-warping is one example of a more sophisticated data analysis method that should be used to advance scientific insights. Our example of manipulating a whip as a testbed for motor control tried to take one step into this direction.

### Controlling the flow of time in motor actions?

It is commonly known that a masterly delivery of a piano piece modulates the regular flow of time by stretching and compressing intervals away from the regular rhythm to enhance the perceived aesthetic quality. In psychology it is known that, counter to the linearly and uniformly evolving external time, its perception may be affected by concurrent mental activity and the sense of agency^52–54^. Another widely known example is that time intervals that were filled with noteworthy events appear longer in memory than those with little content^55,56^. Further, pronouncing a sentence or a word has distinctive features to convey its meaning, but each speaker endows it with a different ‘melody’ or prosody. This temporal stretching or compressing of the defining features can be viewed as controlling the evolution of time. Similar to intonation, stress, and rhythm in speech, the relative timing of action features may be modulated or controlled together with speed, jerk or curvature, features that are typically considered in motor control research. When using time-warping analysis, variable execution rates in different trajectories are streamlined, assuming an underlying common shape of the trajectories. The warping functions reflect the control of time. If movement at the neural level is encoded in terms of time, the time-warping method may get us closer to understanding movement in the way it is controlled.

## Figures and Tables

**Fig. 8:**
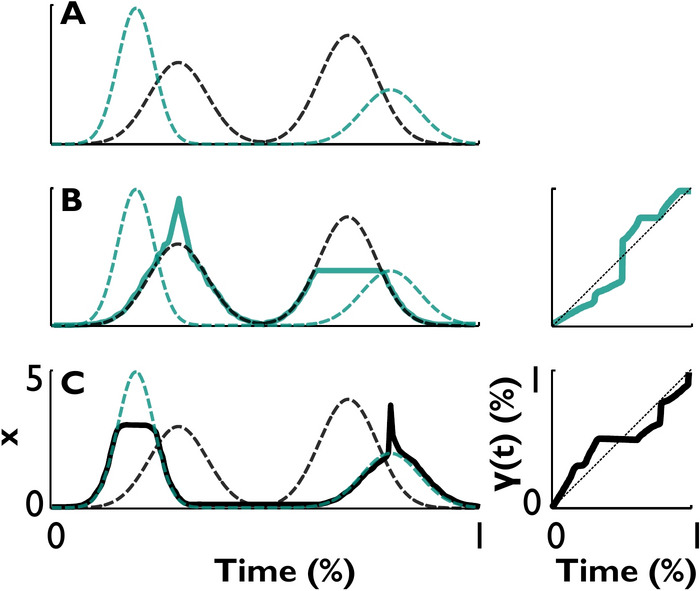
Pinching effect arising in the unconstrained dynamic time-warping (DTW) algorithm. (**A**) Original signals (same as in Fig. 2). (**B**) Green-to-black alignment with its warping function on the right. (**C**) Black-to-green alignment, with its warping function on the right.

**Fig. 9:**
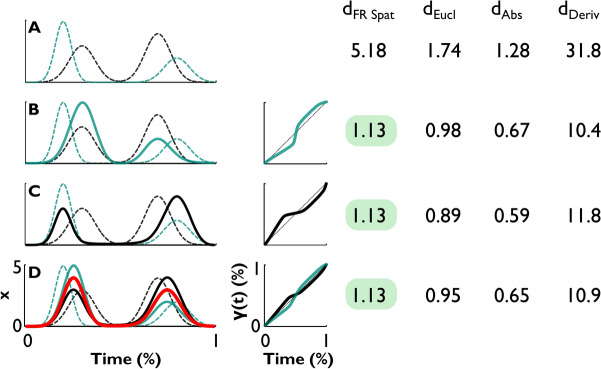
Comparison of four distance metrics applied to aligning a pair of signals in three different ways. (**A**) Two non-aligned signals (same as in Fig. 8). (**B**) Teal-to-black alignment, with its warping function on the right. **C:** Black-to-teal alignment, with its warping function on the right. (**D**) Simultaneous alignment of the two signals, and its warping function. The four distance metrics are the Euclidean distance (L2-norm) between the signals *d*_*Eucl*_, the absolute distance (L1-norm) between the signals *d*_*Abs*_, the L2-norm on the time-derivatives of the signals *d*_*Deriv*_, and the Fisher-Rao metric *d*_*FR*_. The smallest distance per column is marked with green shading. Only the Fisher-Rao metric is invariant across all three alignments.

## Data Availability

All data generated or analyzed during this study are included in this published article. Please see the GitHub repository EFDA_MovementSignalAlignment, at https://github.com/dondestamos/EFDA_MovementSignalAlignment.

## Appendix I: Definition of terminology

As different approaches for time-warping alignment have been developed and used in different research areas, various terminology conventions have been adopted. Below we provide definitions and how we used the terms in this paper.

- The terms *curve, function*, *signal*, and *time-series* are used in a partially interchangeable manner, but they also have some specific contextual meaning. *Function* refers to a formal mathematical mapping, or association, of elements in one domain to (usually) unique elements in another domain. *Curve* in the context of functional data analysis refers to a function that maps a one-dimensional domain onto an n-dimensional one, such as a curve in coordinates XY is two-dimensional and can be parametrized as *x(t)* and *y(t)* for some parameter *t*. Both curves and functions are considered continuous, i.e., consisting of infinitely many elements. The terms *signal* and *time-series*, on the other hand, denote discrete, finite sequences of time-ordered data points resulting from sampled measurements. Software packages, such as the one created in this study, numerically implement a functional algorithm to signals or time-series. When discussing elastic functional data analysis, we use *curve* as the most general term and *signal* or *time-series* as the most measurement-related terms.
- The term *registration* refers to the alignment of one or more curves to a template to match their visually noticeable features, e.g., peaks, valleys, or slopes. Registration may be manual and based on selected landmarks, or continuous including all data samples. Expressed mathematically, continuous registration includes re-parametrization of time, i.e., uniform and non-uniform temporal scaling of time-series, as reviewed here, and may include phase-amplitude separation^45^. Continuous registration requires optimization to minimize a distance metric between the curves, as considered in this study. Registration may also include other methods that facilitate alignment of landmarks, yet do not include temporal scaling, such as Procrustes alignment (translation, rotation, and uniform spatial scaling)^47^.
- The *template* in alignment, or registration, refers to a curve acting as a reference for registration. Often, a signal is pre-defined as the template for quantifying the degree of similarity between the curves. The example study in this paper illustrates the case when the template is the emerging “mean” of the ensemble curves; this is often referred to as Kärcher mean and can be iteratively approximated via EFDA^7^.
- *Re-parametrization* of a curve refers to re-defining the curve in terms of a new independent variable, without altering the shape (or amplitude) of the curve itself. A general parametric curve *β*(*s*) can be re-parametrized with respect to a function *γ*(*s*) to generate *β*(*γ*). To preserve the shape of the curve, the *warping function γ*(*s*) must be smooth, strictly monotonic, and invertible. Time-normalization with *γ*(*s*) = *s/s*_*max*_ is an example of such re-parametrization. When the domain of the new independent variable should be the domain of the original variable, the warping function must be diffeomorphic.
- *Time-warping* is a term used for diffeomorphic re-parametrization of one-dimensional (rarely higher-dimensional) time-series where the original independent variable is normalized time. Numerically and programmatically, time-warping is equivalent to interpolation of a signal from one support to a new one, where either support (or both) may have non-linear spacing.
- *Dynamic time-warping* (DTW) conventionally refers to time-warping by minimizing the sum of Euclidean distances between sequential pairs of signal points, implemented via dynamic programming algorithms. Other distance metrics may be indicated with a prefix, such as derivative-DTW (dDTW) or probabilistic DTW (pDTW)^3,57^. The frequent use of the acronym DTW has resulted in an (incorrect) conflation of the terms “time-warping” and “DTW”.
- *Time-warping alignment* is a term we adopted for the simultaneous alignment of multiple time-series to an emerging common template, i.e., estimated Kärcher mean, minimizing the sum of Fisher-Rao distances between the time-series and the template. Other terms were previously suggested, such as elastic alignment^47^.
- The *Fisher-Rao distance* is a geodesic distance metric minimizing Fisher information on a Riemannian manifold. In the context of curves, a convenient expression of that metric coincides with the L2-norm, or the sum of Euclidean distances between the square-root-rate-function (SRRF) of the curves (see section “Aligning signals: calculating the distance between two or more signals” in the main text and Appendix 2). The Fisher-Rao distance is part of the alignment algorithm used this paper^47^.
- *Time-shift,* or *γ*(*t*) − *t*, refers to a transformed version of a warping function from which identity was subtracted to facilitate examination of temporal transformation. Vertical coordinates of the time-shift reflects how far a point of the warped signal was shifted in time from the original signal (see also^58^ for this use of terminology). Time-shift is similar to the term displacement field^48^, but the latter is defined for *inversed* warping functions, so that a vertical coordinate indicates how far a point of the original signal was shifted in time to produce the warped signal.
- *Spatial* or *amplitude variability* refers to the root-mean-square error (RMSE) across the ensemble of time-series, with the same units as the time-series variable.
- *Temporal variability* is a term we adopted to refer to RMSE across the ensemble of warping functions obtained from the alignment of an ensemble of time-series. The units are dimensionless due to normalized time. Note, some authors use the same term to refer to the contribution of temporal variability to the spatial domain^5,37^.
- *Temporal-to-spatial variability* refers to the contribution of temporal variability to the spatial domain. We adopted this term for the RMSE calculated across an ensemble of time-series that was transformed: the mean of the aligned time-series is warped with every warping function that resulted from alignment. The resulting units are those of the original time-series.

## Appendix II: Different distance metrics

One important aspect in which time warping methods differ is the distance metric used to align the data. One intuitive distance that is used by dynamic time warping (DTW) maps a pair of time-series onto a real number via the Euclidean distance between the signals, i.e., the L2-norm of the difference. Since the seminal paper by Sakoe and Chiba^1^, this distance metric has been widely used for data analysis in speech analysis and gesture recognition and also some biomechanical and clinical studies^3,43,59^. A close alternative of this distance metric is the L1-norm, or Manhattan distance. One potential drawback of both distances is that they are non-symmetric, i.e., their value depends on the direction of alignment^5^. Although special ‘weighting’ may provide an approximation of the symmetry property, its formal absence has remained a concern for theorists.

Another serious drawback of the Euclidean distance metric is ‘pinching’ behavior. Minimizing the sum of those distances may lead to an exact overlay of multiple signal points into one point of the time axis. Figure 8 demonstrates such pinching behavior in two synthetic signals, where warping functions were generated via the *dtw*-function in Matlab. Alignment of the green to the black signal produced a sharp first peak and completely flattened the second peak (Fig. 8B); the inverse occurred when aligning the black to the green signal (Fig. 8C). While such behavior may be compensated by various constraints on the warping function, the choice of those constraints and the resulting dynamic distortion remain challenging to control.

One way to mitigate this pinching behavior is by selecting a metric that places more emphasis on aligning the slopes, rather than single values of the original signals, and that includes an implicit trade-off between alignment and the amount of warping. Following this consideration, the derivative-DTW approach minimizes the Euclidean distance between the time-derivatives of the original signals. As a signal *x*(*t*) is warped with the warping function *γ*(*t*), producing *x*(*t*) ◦ *γ*(*t*) = *x*(*γ*(*t*)), the derivative *x*′(*t*) is not only warped in a similar way, but also vertically rescaled by the derivative of the warping function: *x*′(*t*) ◦ *γ*(*t*) = *x*′(*γ*(*t*)) ∙ *γ*′(*t*). When used for alignment of kinematic signals in human locomotion data, this method was inferior even to the piecewise semi-manual time-normalization^43^.

The distance metric that is used for this tutorial was developed by Srivastava and colleagues ^7,11,60^. It is based on an exponential mapping of each of the original functions to a Riemannian manifold and then finding the Fisher-Rao distance between the mappings. That metric is also conveniently expressed in the original time-series space as Euclidean distance between the signed-square-root-derivatives of the signals. In addition to mitigating pinching, it is the only proper distance metric on a Riemannian manifold between a pair of curves that is invariant to simultaneous warping^12,13^. The latter property is mandatory for many scientific questions where the template cannot be determined a priori.

To illustrate the dependency of the alignment on the distance metric, Fig. 9 repeats the three directions of alignment of two signals via EFDA, first illustrated in Fig. 2. The Fisher-Rao distances in signal space, *d*_*FR*_, were computed. Three additional distance metrics are shown for reference: the Euclidean distance between the signals, *d*_*Eucl*_, the absolute distance (L1-norm), *d*_*Abs*_, and the Euclidean distance between the signal derivatives, *d*_*Deriv*_. The EFDA reduced *d*_*FR*_ from the original signals’ distance (Fig. 9A) to the same minimum value for all three alignment directions. Even though the three other metrics *d*_*Eucl*_, *d*_*Abs*_, and *d*_*Deriv*_ also decreased for the three alignments, their minima differed significantly. This indicates that *d*_*FR*_ captures alignment in a more general way.

Evidently, new metrics and approaches are being developed, but there is still no consensus about the optimal one^25,43,61,62^. The DTW approach with the Euclidean distance remains the most popular choice, although its drawbacks are well-documented. While a choice of metric is often bound to a choice of a mathematical framework and, more practically, to the choice of a computational library, the expected interpretability for a particular dataset should be the main consideration.

## Appendix III: Explanations and comments to the software package

The code supplied with this paper is intended to demonstrate the concept of piecewise and continuous time-warping, i.e., time-warping alignment. It allows to generate figures essentially similar to those provided in the manuscript and equips readers with examples of code usage for further work. The provided repository “EFDA_MovementSignalAlignment”^15^ contains the hand speed data from novice and expert participants (.mat), selected components from the *fdasrvf* v3.4 package^14^ with Windows-compiled *mex*-routines, a few customized dependency functions, and the following primary functions:

- *EFDA_warps_visual_continuous_asymm*: Visualizes the effect of time-warping on an exemplary 2-peaked signal when warping functions are entirely below or above the identity (*γ* = *t*).
- *EFDA_warps_visual_continuous_symm*: Visualizes effect of time-warping on a signal when warping functions are both below and above the identity.
- *EFDA_warps_visual_piecewise*: Visualizes effect of piecewise linear time-warping on a signal.
- *genwarp*: Generates a warping function using a sine-based shooting vector of specified amplitude, frequency, and phase. Optionally, add two kinds of noise (see comments on functions above).
- *EFDA_conceptDemos:* Demonstrates three kinds of alignment of a pair of double-Gaussian signals, displays distance metrics; optionally displays SRRF transforms and emulates L2-minimization and DTW approach.
- *EFDA_GaussModel:* Generates N = 500 noisy realizations of triple-peaked Gaussian signals, uses three methods of resampling and alignment, extracts mean, and evaluates the accuracy of landmark characterization.
- *EFDA_WhipAverage_ShowVar*(data, SubjType, AverMethod): Uses the data provided in the repository to align, average, and characterize a participant’s hand speed via one of four methods discussed in the paper.
- *EFDA_AlignmentMain*(X,varargin): Supplies an arbitrary dataset of multiple time-series for time-warping alignment via EFDA. Uses a wide range of custom options for quick preview, performance/accuracy balance etc.
- *fdawarp*: a class from the package *fdasrvf* (v3.4) from which we used the method *time_warping* for time-warping alignment of an ensemble to an emerging “Kärcher mean” template. For a given ensemble of signals, such template is found via converging iterations. At each iteration, a mean signal is first estimated, then corresponding warping functions from original ensemble to that mean are found via a dynamic programming-implemented minimization of pairwise *d*_*FR*_ (see the function *optimum_reparam.m*). Then, the ensemble is updated to its “aligned” version which will be used during the next iteration (if any). More details are provided in the original works introducing EFDA^7,11,14^. Our edits in that class and method include outputting the amplitude and temporal distances between the aligned time-series and their mean, and retaining the data from all iterations of the alignment.
- *warp_f_gamma*: implements time-warping, or re-parametrization, of time-series, in the *fdasrvf* package and in our software. For numerical purposes, time-warping is equivalent to one-dimensional interpolation, hence the built-in function *interp1* was used.

## References

[1] Sakoe, H. & Chiba, S. Dynamic programming algorithm optimization for spoken word recognition. *IEEE Trans. Acoust.* **26**, 43–49 (1978).

[2] Bruderlin, A. & Williams, L. Motion signal processing. In *Proceedings of the 22nd Annual Conference on Computer Graphics and Interactive Techniques* 97–104 (1995).

[3] Bautista, M. Á. et al. Probability-based dynamic time warping for gesture recognition on RGB-D data. *Lect. Notes Comput. Sci. Artif. Intell. Bioinform.* **7854**, 126–135 (2013).

[4] Ramsay, J. O. & Silverman, B. W. *Functional Data Analysis* (Springer, New York, 2005).

[5] Srivastava, A. & Klassen, E. P. *Functional and Shape Data Analysis* (Springer, New York, 2016).

[6] Boulgouris, N. V., Plataniotis, K. N. & Hatzinakos, D. Gait recognition using dynamic time warping. In *2004 IEEE 6th Workshop on Multimedia Signal Processing* 263–266 (2004).

[7] Tucker, J. D., Wu, W. & Srivastava, A. Generative models for functional data using phase and amplitude separation. *Comput. Stat. Data Anal.* **61**, 50–66 (2013).

[8] Kurtek, S., Srivastava, A., Klassen, E. & Ding, Z. Statistical modeling of curves using shapes and related features. *J. Am. Stat. Assoc.* **107**, 1152–1165 (2012).

[9] Lee, J. et al. Functional data analyses of gait data measured using in-shoe sensors. *Stat. Biosci.* **11**, 288–313 (2019). 32426061 10.1007/s12561-018-9226-3 PMC7233106

[10] Wu, W. & Srivastava, A. An information-geometric framework for statistical inferences in the neural spike train space. *J. Comput. Neurosci.* **31**, 725–748 (2011). 21584775 10.1007/s10827-011-0336-x

[11] Srivastava, A., Wu, W., Kurtek, S., Klassen, E. & Marron, J. S. Registration of functional data using Fisher-Rao metric. arXiv:1103.3817 (2011).

[12] Bruveris, M. Optimal reparametrizations in the square root velocity framework. *SIAM J. Math. Anal.* **48**, 4335–4354 (2016).

[13] Cencov, N. Statistical decision rules and optimal inference. *Transl. Math. Monogr.* **53**, 510 (1982).

[14] Tucker, J.D. Repository fdasrvf by tetonedge at https://github.com/jdtuck/fdasrvf_MATLAB/releases/tag/3.6.3. *Github* (2024).

[15] Krotov, A. Repository EFDA_MovementSignalAlignment by dondestamos at https://github.com/dondestamos/EFDA_MovementSignalAlignment. *Github *(2024).

[16] Nah, M. C., Krotov, A., Russo, M., Sternad, D. & Hogan, N. Learning to manipulate a whip with simple primitive actions—A simulation study. *iScience* **26**, 107395 (2023). 37554449 10.1016/j.isci.2023.107395 PMC10405071

[17] Krotov, A., Russo, M., Nah, M., Hogan, N. & Sternad, D. Motor control beyond reach—How humans hit a target with a whip. *R. Soc. Open Sci.* **9**, 220581 (2022). 36249337 10.1098/rsos.220581 PMC9533004

[18] Edraki, M., Lokesh, R., Krotov, A., Ramezani, A. & Sternad, D. Human-inspired control of a whip: Preparatory movements improve hitting a target. In *IEEE BIOROB* (2024). 10.1109/biorob60516.2024.10719792 PMC11715529 39791118

[19] Nah, M. C., Krotov, A., Russo, M., Sternad, D. & Hogan, N. Dynamic primitives facilitate manipulating a whip. In *2020 8th IEEE RAS/EMBS International Conference for Biomedical Robotics and Biomechatronics (BioRob)* 685–691 (IEEE Computer Society, 2020).

[20] Maselli, A. et al. Beyond simple laboratory studies: Developing sophisticated models to study rich behavior. *Phys. Life Rev.* **46**, 220–244 (2023). 37499620 10.1016/j.plrev.2023.07.006

[21] Stergiou, N. & Decker, L. M. Human movement variability, nonlinear dynamics, and pathology: Is there a connection?. *Hum. Mov. Sci.* **30**, 869–888 (2011). 21802756 10.1016/j.humov.2011.06.002 PMC3183280

[22] Sternad, D. It’s not (only) the mean that matters: Variability, noise and exploration in skill learning. *Curr. Opin. Behav. Sci.* **20**, 183–195 (2018). 30035207 10.1016/j.cobeha.2018.01.004 PMC6051545

[23] Ancillao, A., Savastano, B., Galli, M. & Albertini, G. Three dimensional motion capture applied to violin playing: A study on feasibility and characterization of the motor strategy. *Comput. Methods Programs Biomed.* **149**, 19–27 (2017). 28802327 10.1016/j.cmpb.2017.07.005

[24] Haar, S., van Assel, C. M. & Faisal, A. A. Motor learning in real-world pool billiards. *Sci. Rep.* **10**, 1–13 (2020). 33208785 10.1038/s41598-020-76805-9 PMC7674448

[25] Raket, L. L., Grimme, B., Schöner, G., Igel, C. & Markussen, B. Separating timing, movement conditions and individual differences in the analysis of human movement. *PLOS Comput. Biol.* **12**, e1005092 (2016). 27657545 10.1371/journal.pcbi.1005092 PMC5033575

[26] Swaminathan, K. et al. A continuous statistical-geometric framework for normative and impaired gaits. *J. R. Soc. Interface* **19**, 20220402 (2022). 36321374 10.1098/rsif.2022.0402 PMC9627451

[27] Henrot, C. Characterization of whip targeting kinematics in discrete and rhythmic tasks. *Bachelor of Science Thesis* (Massachusetts Institute of Technology, 2016).

[28] Krotov, A. *et al.* Manipulating a bullwhip—simple control of a complex object? In Proceedings *The 9.5th International Symposium on Adaptive Motion of Animals and Machines. Ottawa**, **Canada (Virtual Platform).* 58 -59 (2021).

[29] Rath, T. M. & Manmatha, R. Word image matching using dynamic time warping. In *Proceedings of the IEEE Computer Society Conference on Computer Vision and Pattern Recognition* Vol. 2 (2003).

[30] Park, C., Do-Noh, S. & Srivastava, A. Data science for motion and time analysis with modern motion sensor data. *Oper. Res.* **70**, 3217–3233 (2022).

[31] Ben Amor, B., Srivastava, A., Turaga, P. & Coleman, G. A framework for interpretable full-body kinematic description using geometric and functional analysis. *IEEE Trans. Biomed. Eng.* **67**, 1761–1774 (2020). 31603769 10.1109/TBME.2019.2946682

[32] Dhawale, A. K., Smith, M. A. & Ölveczky, B. P. The role of variability in motor learning. *Annu. Rev. Neurosci.* **40**, 479–498 (2017). 28489490 10.1146/annurev-neuro-072116-031548 PMC6091866

[33] Krotov, A. EFDA Movement Data. *Github* (2024).

[34] Velichko, V. M. & Zagoruyko, N. G. Automatic recognition of 200 words. *Int. J. Man. Mach. Stud.* **2**, 223–234 (1970).

[35] Sederberg, T. W. & Greenwood, E. A physically based approach to 2–D shape blending. *ACM SIGGRAPH Comput. Graph.* **26**, 25–34 (1992).

[36] Kneip, A. & Gasser, T. Statistical tools to analyze data representing a sample of curves. *Ann. Stat.* **20**, 1266–1305 (1992).

[37] Kneip, A. & Ramsay, J. O. Combining registration and fitting for functional models. *J. Am. Stat. Assoc.* **103**, 1155–1165 (2008).

[38] Troje, N. F. Decomposing biological motion: A framework for analysis and synthesis of human gait patterns. *J. Vis.* **2**, 2–2 (2002). 10.1167/2.5.2 12678652

[39] Russo, A. A. et al. Neural trajectories in the supplementary motor area and motor cortex exhibit distinct geometries, compatible with different classes of computation. *Neuron* **107**, 745–758 (2020). 32516573 10.1016/j.neuron.2020.05.020 PMC9395139

[40] Arribas-Gil, A. & Müller, H. G. Pairwise dynamic time warping for event data. *Comput. Stat. Data Anal.* **69**, 255–268 (2014).

[41] Moudy, S., Richter, C. & Strike, S. Landmark registering waveform data improves the ability to predict performance measures. *J. Biomech.* **78**, 109–117 (2018). 30126719 10.1016/j.jbiomech.2018.07.027

[42] Zin, M. A., Rambely, A. S. & Ariff, N. M. Effectiveness of landmark and continuous registrations in reducing inter-and intrasubject phase variability. *IEEE Access* **8**, 216003–216017 (2020).

[43] Helwig, N. E., Hong, S., Hsiao-Wecksler, E. T. & Polk, J. D. Methods to temporally align gait cycle data. *J. Biomech.* **44**, 561–566 (2011). 20887992 10.1016/j.jbiomech.2010.09.015

[44] Dreyfus, S. E. & Bellman, R. E. *Applied Dynamic Programming* (Princeton University Press, Princeton, 1962).

[45] Marron, J. S., Ramsay, J. O., Sangalli, L. M. & Srivastava, A. Functional data analysis of amplitude and phase variation. *Stat. Sci.* **30**, 468–484 (2015).

[46] Guo, X., Wu, W. & Srivastava, A. Data-driven, soft alignment of functional data using shapes and landmarks. arXiv:2203.14810 (2022).

[47] Srivastava, A., Klassen, E., Joshi, S. H. & Jermyn, I. H. Shape analysis of elastic curves in euclidean spaces. *IEEE Trans. Pattern Anal. Mach. Intell.* **33**, 1415–1428 (2011). 20921581 10.1109/TPAMI.2010.184

[48] Pataky, T. C., Robinson, M. A., Vanrenterghem, J. & Donnelly, C. J. W. Simultaneously assessing amplitude and temporal effects in biomechanical trajectories using nonlinear registration and statistical nonparametric mapping. *J. Biomech.* **136**, 111049 (2022). 35430435 10.1016/j.jbiomech.2022.111049

[49] Wang, Q. Computational modeling and analysis of symmetry in human movements. A Ph.D. Thesis (Arizona State University, 2018).

[50] Maselli, A. et al. A whole body characterization of individual strategies, gender differences, and common styles in overarm throwing. *J. Neurophysiol.* **122**, 2486–2503 (2019). 31577474 10.1152/jn.00011.2019

[51] Zhang, Z. & Sternad, D. Back to reality: Differences in learning strategy in a simplified virtual and a real throwing task. *J. Neurophysiol.* **125**, 43–62 (2021). 33146063 10.1152/jn.00197.2020 PMC8087380

[52] D’Agostino, O., Castellotti, S. & Del Viva, M. M. Time estimation during motor activity. *Front. Hum. Neurosci.* **17**, 172 (2023). 37151903 10.3389/fnhum.2023.1134027 PMC10160443

[53] Haggard, P., Clark, S. & Kalogeras, J. Voluntary action and conscious awareness. *Nat. Neurosci.* **5**, 382–385 (2002). 11896397 10.1038/nn827

[54] Muhs, K. S., Karwowski, W. & Kern, D. Temporal variability in human performance: A systematic literature review. *Int. J. Ind. Ergon.* **64**, 31–50 (2018).

[55] Hass, J. & Durstewitz, D. Time at the center, or time at the side? Assessing current models of time perception. *Curr. Opin. Behav. Sci.* **8**, 238–244 (2016).

[56] Angrilli, A., Cherubini, P., Pavese, A. & Manfredini, S. The influence of affective factors on time perception. *Percept. Psychophys.* **59**, 972–982 (1997). 9270369 10.3758/bf03205512

[57] Keogh, E. J. & Pazzani, M. J. Derivative dynamic time warping. In *Proceedings of the 2001 SIAM International Conference on Data Mining* 1–11 (Society for Industrial & Applied Mathematics (SIAM), 2001).

[58] Giese, M. A. & Poggio, T. Synthesis and recognition of biological motion patterns based on linear superposition of prototypical motion sequences. In *Proceedings IEEE Workshop on Multi-View Modeling and Analysis of Visual Scenes (MVIEW’99)* 73–80 (1999).

[59] Weiske, F., Böhme, M., Jäkel, J., Zentner, J. & Witt, M. Stair ascent comparison of lower limb kinematics with differing time normalization techniques. *J. Biomech.* **119**, 110316 (2021). 33631663 10.1016/j.jbiomech.2021.110316

[60] Veeraraghavan, A., Srivastava, A., Roy-Chowdhury, A. K. & Chellappa, R. Rate-invariant recognition of humans and their activities. *IEEE Trans. Image Process.* **18**, 1326–1339 (2009). 19398409 10.1109/TIP.2009.2017143

[61] Haji Ghassemi, N. et al. Segmentation of gait sequences in sensor-based movement analysis: a comparison of methods in Parkinson’s disease. *Sensors* **18**, 1–15 (2018). 10.3390/s18010145 PMC5796275 29316636

[62] Carroll, C. & Müller, H.-G. Latent deformation models for multivariate functional data and time-warping separability. *Biometrics* (2023). 36877941 10.1111/biom.13851 PMC10480349

